# Technical Criteria for Delimiting the Picanha Beef Cut: A Preliminary Study

**DOI:** 10.1111/1750-3841.71424

**Published:** 2026-09-04

**Authors:** Isabela Benfica de Barros, Jonatã Henrique Rezende‐de‐Souza, Dyana Carla Lima Hargreaves Noguera, Gabriel Barão Arias Blanco, Cíntia Rizoli, Sílvio Roberto Consonni, Sérgio Bertelli Pflanzer

**Affiliations:** ^1^ Department of Engineering and Food Technology, School of Food Engineering University of Campinas Campinas São Paulo Brazil; ^2^ Department of Biochemistry and Tissue Biology, Institute of Biology University of Campinas Campinas Brazil

**Keywords:** barbecue, beef cut, histological analysis, outside round, sirloin cap, third vein

## Abstract

The Picanha, obtained from the *Biceps femoris* muscle, is highly valued worldwide. No official standard precisely defines this cut, leading slaughterhouses to rely on subjective criteria such as weight, length, or the location of the “third vein”. Thus, this study aimed to characterize variations along the entire length of the *Biceps femoris* muscle to identify the location of the separation of Picanha. Four *Biceps femoris* muscles were sectioned into 12 consecutive steaks (2.5 cm each) along the proximal–distal axis and subjected to physicochemical and histological analyses. Between steaks 6 and 7, the average weight of the cut (∼1.5 kg) was consistent with empirical industry standards, and the “third vein” was identified at steak 6, reinforcing its practical relevance. A decrease in pH was observed along the proximal–distal axis (5.79 to 5.62; *P* < 0.05). Total fat decreased toward the distal region (*P* < 0.01), whereas total moisture and collagen content increased (*P* < 0.05 and *P* < 0.001, respectively). Sarcomere length exhibited linear and quadratic effects (*P* < 0.001 and *P* < 0.05, respectively), with greater values in proximal steaks and progressive shortening toward distal regions. Similarly, Warner–Bratzler shear force showed a quadratic increase (*P* < 0.01), with steaks 1–6 classified as tender (≤ 4.4 kg) and steaks 7–12 as non‐tender. Histological evaluation confirmed structural differences consistent with these findings. Overall, the transition in physicochemical and structural traits occurred around steaks 6–7, corresponding to the location of the “third vein.” These results support its use as a practical and biologically meaningful anatomical reference for delimiting the Picanha cut.

**Practical Applications:**

The anatomical delimitation of the *Biceps femoris* muscle was evaluated under controlled experimental conditions using steaks of standardized thickness, which may differ from commercial fabrication practices. The results consistently identified physicochemical and structural transitions around the “third vein”, supporting its use as a practical anatomical reference for standardizing the Picanha (sirloin cap or rump cap) and Coxão Duro (outside round or bottom round) cuts in the meat industry and gastronomy.

## 1 Introduction

Beef plays a central role in culinary traditions worldwide, not only as a source of nutrients but as a cultural symbol embedded in social and celebratory practices. Barbecue represents one of the most emblematic gastronomic practices, where specific cuts are associated with social status, sensory expectations, and culinary expertise (Bonfim et al. 2024; Cacenot 2024; Sabatini et al. 2025). Among these, Picanha, commercially known as sirloin cap or rump cap, is regarded as a premium cut and occupies a privileged position in Brazilian barbecue culture, ranked as the 15th best dish in the world in 2026, according to the TasteAtlas Awards (2026). Its prestige is linked to its distinctive fat cover, characteristic flavor, and perceived tenderness, attributes that shape consumer preference and justify its higher market value (Beyer et al. 2021).

Picanha is obtained from the proximal portion of the *Biceps femoris* muscle, which also gives rise to Coxão Duro (outside round or bottom round) in its distal region. Although both cuts originate from the same muscle, they differ markedly in culinary application, sensory perception, and commercial valuation (Rhee et al. 2004; Sullivan and Calkins 2011). In the Brazilian market, Picanha typically costs at least two more than the price of Coxão Duro, reflecting not only structural differences but also cultural recognition and gastronomic demand. This disparity highlights the importance of understanding how anatomical and structural variation within a single muscle translates into differentiated eating experiences and economic value.

From a biological perspective, the *Biceps femoris* extends along the posterior region of the bovine hindquarter and exhibits gradual structural changes along its proximal–distal axis. Variations in connective tissue distribution, total intramuscular fat deposition, sarcomere length, and collagen characteristics can directly influence tenderness, juiciness, and overall palatability of the beef, attributes that are central to gastronomic appreciation (Rhee et al. 2004; Sullivan and Calkins 2011).

Despite its cultural and economic relevance, the meat sector lacks an officially standardized anatomical definition for Picanha. According to the Technical Regulation of Identity and Quality of Meat Cuts (Brazil—Ministério da Agricultura 1988), Picanha is defined as a subdivision of the sirloin, located in the dorsal portion of the bovine hindquarter. This regulation states that the cut is obtained during the deboning of the sirloin, through the separation of the connective tissue uniting the *Biceps femoris* and *gluteus medius* muscles, and is characterized by a thick external fat layer, commonly referred to as the “fat cap,” covering its upper surface.

In practice, slaughterhouses rely on empirical criteria such as cut weight, length of the piece, or the position of a visible vascular structure commonly referred to in the industry as the “third vein”. Although not formally recognized in anatomical terminology, this “vein” serves as a practical landmark used to separate the proximal region (commercially identified as Picanha) from the distal portion associated with Coxão Duro. However, because this criterion is applied inconsistently across processing plants, variability arises in cut delimitation, potentially affecting product authenticity, sensory consistency, labeling practices, and consumer expectations.

In a gastronomic context, such variability is particularly relevant. When a premium cut is defined primarily by tradition and sensory identity rather than by precise anatomical boundaries, inconsistencies in its delimitation may lead to fluctuations in tenderness, fat content, and cooking performance. These variations can compromise the eating experience and weaken the alignment between cultural expectation and actual product quality. Therefore, establishing objective and biologically grounded criteria for defining Picanha may contribute not only to industrial standardization but also to culinary authenticity and consumer trust. Given this scenario, the present study aims to characterize, in a preliminary and controlled manner, the structural, histological, compositional, and instrumental tenderness variations along the entire length of the *Biceps femoris* muscle, from its proximal to distal regions, in order to identify the distinction between the commercial cuts known as Picanha (sirloin cap or rump cap) and Coxão Duro (outside round).

## 2 Materials and Methods

### 2.1 Sample Preparation

Eight whole *Biceps femoris* muscles from the right and left were collected from four uncastrated *Bos taurus indicus*, Nellore breed, approximately 36 months of age, slaughtered in a Brazilian federally inspected slaughterhouse. The muscles comprised the proximal Picanha (sirloin cap or rump cap) and distal Coxão Duro (outside round or bottom round) portions. Cuts were identified from the right and left sides of each carcass. Muscles were collected during deboning at 48 h postmortem, vacuum‐packaged, placed in insulated boxes with ice, and transported to the laboratory.

**FIGURE 1:**
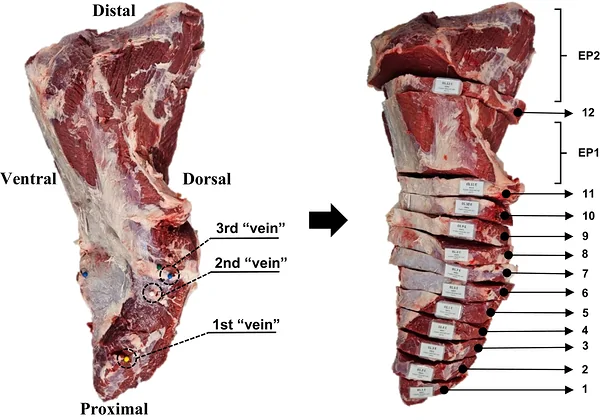
Location of “veins” in *Biceps femoris* muscle and steak samples. *Note*: Steak position: starting from the proximal end (1) toward the distal end (12). EP: Excluded portion (distal muscle portion excluded from the quality analyses because it belonged entirely to the outside round region and was not considered part of the anatomical transition between Picanha and Coxão Duro).

Each muscle was oriented from the proximal region (Picanha) to the distal region (Coxão Duro). Visible vascular landmarks (veins) were identified using pins before sectioning. The proximal end of each *Biceps femoris* muscle was first sectioned into a 5‐cm‐thick steak. This thickness was intentionally adopted based on preliminary laboratory trials demonstrating that a 2.5‐cm steak from this anatomical region did not provide sufficient sample mass for all planned physicochemical, structural, histological, and instrumental analyses. Subsequently, the remaining muscle was sectioned into consecutive 2.5‐cm‐thick steaks along the proximal–distal axis. After steak 11, a distal segment equivalent to four consecutive steaks (10 cm) was intentionally excluded because it represented a region unequivocally identified as Coxão Duro and therefore did not contribute to the objective of identifying the anatomical transition between Picanha and Coxão Duro. Following the removal of this distal segment, one final 2.5‐cm steak (steak 12) was collected to represent the standardized distal portion of the muscle, and the remaining tissue was discarded. All analyzed steaks were numbered sequentially according to their anatomical position along the *Biceps femoris* muscle, as illustrated in Figure 1.

In the steaks from the left side, each one was weighed, individually vacuum‐packed, frozen at –18°C, and stored until the cooking loss and shear force analyses were performed. In the steaks from the right side, they were directly vacuum‐packed, frozen at –18°C, and stored until the analyses of pH, total moisture and fat content, sarcomere length by laser and microscopy, and collagen content were performed. For histological analysis, fragments were taken from the right‐side steaks, placed in histological cassettes, and kept in a refrigerated formalin solution until the histological analyses were conducted.

Because the primary objective of the study was to identify the anatomical transition between Picanha and Coxão Duro, variables directly related to instrumental tenderness, including Warner–Bratzler shear force (WBSF), pH, and sarcomere length, were evaluated in all steaks along the *Biceps femoris* muscle. WBSF was selected as the principal response variable because it is the only instrumental method with internationally recognized tenderness classifications established by the American Society for Testing and Materials (ASTM 2011). Sarcomere length and pH were also determined throughout the muscle because both are closely associated with the biological mechanisms underlying meat tenderness. In contrast, compositional analyses (moisture, fat, and collagen) and histological analyses were performed on representative anatomical regions to characterize changes in muscle composition along the proximal–distal axis while maintaining the feasibility of laboratory analyses.

### 2.2 Steak Weight

All steaks obtained from the left‐side muscles were individually weighed to evaluate the influence of cumulative weight on the practical separation between Picanha and Coxão Duro. Steak weights were recorded sequentially, and cumulative weight was calculated as each new steak from the same animal was weighed, proceeding from the proximal to the distal portion until the entire *Biceps femoris* muscle had been processed.

### 2.3 PH, Total Moisture, and Fat, and Collagen Analyses

For pH, total moisture and fat, and collagen analyses, the external subcutaneous fat was removed from each selected steak, and only the muscle tissue was used. Samples were individually ground using a food processor (Retsch, GRINDOMIX GM 200, Germany), and aliquots of the homogenized meat were distributed according to the requirements of each analysis.

pH was measured in steaks 1 through 12 from the right side. For this procedure (Zenebon et al. 2008), 10 g of sample were diluted in 100 mL of distilled water, homogenized, and analyzed using a bench pH meter (Ohaus, Starter 2100, USA) previously calibrated with standard buffer solutions (pH 4.0 and 7.0). Three readings were taken for each sample.

Total moisture content was determined in steaks 2, 5, and 10 from the right side by the gravimetric method described by (AOAC 1980). 10 g of sample were weighed in pre‐dried and tared Petri dishes and dried in a forced‐air oven at 105°C (Fanem do Brasil S.A., model 315‐SE, Brazil) for 12 h. After cooling in a desiccator, samples were reweighed, and moisture percentage was calculated.

Total fat content was determined using the dried samples from steaks 2, 5, and 10 obtained from the moisture analysis, according to the method of Bligh and Dyer (1959). A total of 1.5 g of dried sample was subjected to extraction using methanol and chloroform to form a monophasic system, followed by phase separation with additional chloroform and distilled water. The lipid‐containing chloroform phase was isolated, the solvent was evaporated, and the remaining lipid residue was gravimetrically quantified. The analysis was performed in duplicate for steak.

Collagen fractions (total, soluble, and insoluble) were determined in steaks 2, 5, 7, and 10 from the right side, using hydroxyproline quantification adapted from Woessner (1961) and Cross et al. (1973). A total of 4 g of samples subjected to buffered extraction, heated in a water bath at 80°C for 60 min, cooled on ice, centrifuged at 13,000 rpm (Cência, Xiangyi, China), and separation of soluble and insoluble fractions. Both fractions were hydrolyzed in 6 N HCl at 110°C for 48 h. After cooling, activated charcoal was added to the samples (300 mg for the supernatant and 900 mg for the residue), followed by filtration and pH adjustment to between 6.5 and 7.0 using 2 N NaOH (Sodium Hydrxide) or 1 N HCl (Hydrochloric Acid). The samples were then transferred to volumetric flasks (100 mL for the supernatant and 250 mL for the residue). Next, the hydroxyproline colorimetric reaction was carried out by adding isopropanol, oxidizing solution, and Ehrlich's reagent. The samples were incubated in a water bath at 60°C for 25 min and cooled on ice. Finally, absorbance readings were performed using a glass cuvette in a spectrophotometer (Orion, AquaMate 8000, Brazil) at 558 nm for collagen quantification. The analysis was performed in duplicate for steak.

### 2.4 Sarcomere Length by Laser and Contrast Microscopy

Sarcomere length was evaluated in steaks 1 to 12 from the right side, using both laser diffraction and phase‐contrast microscopy (Battaglia et al. 2019). For laser diffraction, muscle samples into dimensions of 2 × 2 × 1 cm were fixed in a 5% glutaraldehyde solution, prepared with 0.1 M Na_2_HPO_4_ (Disodium Phosphate) at pH 7.2, at 10°C for 24 h. After, the samples were washed in 0.2 M sucrose solution, and ten small fragments of muscle fibers were separated, placed on microscope slides with a drop of sucrose, and covered with a coverslip. The analysis was performed using a helium–neon laser beam device (Spectra Physics Inc., 117A, USA) with a wavelength of 632.8 nm. The myofibrils were positioned under the beam, and the diffraction pattern was measured using a caliper. For each slide, ten different measurements were taken, and the values obtained were converted to micrometers. For phase‐contrast microscopy, approximately 1 g of muscle sample was homogenized in 10 mL of 0.25 M sucrose solution at 4°C, using a homogenizer (IKA, Ultra Turrax T10, Germany) at 15,000 rpm for 15 to 30 seconds. A drop of the suspension was placed on slides, covered with a coverslip, and observed under a phase‐contrast microscope (Nikon, Eclispe Si, Japan). Ten myofibril fragments per slide were measured using AxioVision L.E. software (Version 4.8). The mean sarcomere length was obtained by dividing the total measured distance by the number of sarcomere units present in the filament.

### 2.5 Cooking Loss and Warner‐Bratzler Shear Force

The procedures followed the methodology outlined by the American Meat Science Association (AMSA 2015) on steaks 1 to 12 from the left side. Each steak was weighed and cooked in a conventional oven (Fritomaq, model 90 × 90, Brazil) at 180°C, with a thermocouple inserted at its geometric center. When the internal temperature reached 45°C, the steak was flipped to complete cooking, which was considered finished when the internal temperature reached 71°C. The steak was removed from the oven and weighed again to obtain the final weight. The steaks were cooled at 4°C for 24 h and used for shear force analysis. To do this, six cores, each 1.27 cm in diameter, were extracted from each steak using a coring cutter parallel to the direction of the muscle fibers. Each core was sheared using a 1 mm‐thick V‐notched Warner–Bratzler blade coupled to a TA‐XT texture analyzer (Stable Micro Systems, Surrey, UK), equipped with a 50‐kg load cell. The instrument operated in compression mode, with a test speed of 3.7 mm/s, a post‐test speed of 40.0 mm/s, and a target mode set to distance (20 mm). Shear force values were recorded in kilograms (kg), and the mean value of the six cores was used for statistical analysis.

### 2.6 Histology With Masson's Trichome Staining

For the histological analysis, all steaks at the right side were evaluated; however, the most noticeable changes were observed in steaks 3, 8, and 10, which were selected for the discussion of the results. For this analysis, a fragment was removed transversely to the fibers from the central region of each steak. Each fragment was placed in histological cassettes, which were inserted into a 10% formalin fixation solution and kept under refrigeration until analysis. After fixation, the tissues were embedded in paraffin and sectioned using a microtome, an instrument with a fixed blade that can be adjusted to different thicknesses, allowing the material to move vertically in a uniform and controlled manner. After sectioning and mounting on microscope slides, the tissue was stained using specific reagents for Masson's trichrome staining. The slides were then mounted with resin and a coverslip to preserve them for observation under a light microscope (Caputo et al. 2010). These slides were used to examine, describe, and characterize the muscle fibers, as well as the fat and connective tissue structures, in samples obtained from different regions of the bovine Biceps femoris muscle, based on observations made from images captured using the Proview software.

### 2.7 Statistical Analysis

Data were analyzed in RStudio Software (version 4.5.2, New Zealand). Descriptive statistics (mean, standard deviation, minimum, and maximum values) were calculated for steak weight data. A repeated‐measures linear mixed‐effects model was used because multiple observations (steaks) were obtained from each animal, resulting in a hierarchical data structure with non‐independent measurements. Steak position along the proximal–distal axis was treated as a fixed effect, whereas animal was included as a random effect to account for the correlation among repeated observations within the same individual and to partition the between‐animal variability from the within‐animal variation attributable to steak position. This approach reduces the risk of pseudoreplication and provides more reliable estimates of the fixed effect under repeated‐measures designs (Lazic 2010; Olsen 2004; Pinheiro and Bates 2020). Data were analyzed using a one‐way repeated‐measures ANOVA (*α =* 0.05). Assumptions of normality and homogeneity of variances were assessed, and analysis of variance (ANOVA) was performed, followed by Tukey's post hoc test for pairwise comparisons. pH, laser sarcomere length, microscopic sarcomere length, cooking loss, and Warner–Bratzler shear force (WBSF) were evaluated in all 12 steaks from each animal because these variables were used to characterize the continuous tenderness profile along the *Biceps femoris* muscle. Total moisture and fat contents were measured in steaks 2, 5, and 10, representing the proximal, intermediate, and distal anatomical regions, respectively. Total, soluble, and insoluble collagen were analyzed in steaks 2, 5, 7, and 10; steak 7 was included exclusively for collagen assessment as an intermediate anatomical region of the muscle. To further investigate the effect of steak position on pH, sarcomere length (laser and microscopic), cooking loss, and WBSF, orthogonal polynomial contrasts were applied to assess linear and quadratic trends across the 12 steak positions. Pearson's correlation coefficient was performed using data from steaks 2, 5, and 10 (four replicates per position) to explore relationships among physicochemical and structural variables.

## 3 Results and Discussion

### 3.1 Steak Weight

In the meat market, the weight of the piece of beef is commonly used by both consumers and industry professionals as a practical criterion to define Picanha. Based on this empirical approach, the cumulative weight of sequential steaks can be used to estimate the extent of the Picanha portion along the *Biceps femoris* muscle. A widely accepted perception is that Picanha should not exceed approximately 1.5 kg, as higher weights are often associated with the inclusion of the distal portion (Coxão Duro). In the present study, this threshold was reached between steaks 6 and 7 in most carcasses (Table 1), indicating that the commonly adopted weight‐based criterion roughly corresponds to the transition between proximal and intermediate regions of the muscle. However, it is important to note that carcass weight can lead to differences in weight for the same muscle cut; thus, larger animals may yield heavier cuts of meat. In addition to the 1.5 kg threshold, another empirical reference used by butchers to distinguish Picanha from Coxão Duro is the presence of the third “vein”. In this context, the anatomical locations of the first, second, and third “veins” consistently corresponded to steaks 2, 5, and 6, respectively, in all evaluated *Biceps femoris* muscles (Table S1). Although the absolute distance from the proximal end of the muscle to each vascular landmark may vary among carcasses due to differences in muscle size and carcass conformation, their anatomical position relative to the standardized steak sequence remained unchanged. Considering the third “vein” as the empirical landmark traditionally used by the meat industry to separate Picanha from Coxão Duro, the transition consistently occurred at the sixth steak under the standardized slicing procedure adopted in this study (steak 1: 5.0 cm; and steaks 2 to 6: 2.5 cm each).

**TABLE 1:** Individual and accumulated weights of steaks from the *Biceps femoris* muscle.

Steak ^A^	Individual steak (g)				Accumulated portion (g)			
	Mean	SD^B^	Min^C^	Max^D^	Mean	SD^B^	Min^C^	Max^D^
1	192	62.0	120	262	192	62.0	120	262
2	172	22.8	139	191	364	73.0	299	442
3	190	24.3	159	218	553	77.6	463	633
4	216	32.2	177	255	769	91.0	640	841
5	255	43.1	208	313	1024	117	848	1,090
6	281	45.7	246	343	1,305	145	1,095	1,427
7	319	47.0	284	388	1,624	181	1,379	1,815
8	389	59.0	350	475	2,012	229	1,730	2,290
9	406	29.8	379	448	2,418	247	2,134	2,738
10	471	27.1	433	498	2,889	258	2,614	3,235
11	523	37.8	473	556	3,412	281	3,130	3,791
EP1	2,014	353	1,557	2,356	5,425	599	4,687	6,146
12	605	48.3	549	667	6,030	647	5,235	6,813
EP2	1,884	361	1,616	2,415	7,914	985	6,851	9,228

*Note*: ^A^Starting from the proximal end (steak 1) toward the distal end (steak 12); ^B^Standard error; ^C^Minimum, ^D^Maximum; (distal muscle portion excluded from the quality analyses because it belonged entirely to the outside round region and was not considered part of the anatomical transition between Picanha and Coxão Duro).

Abbreviation: EP, Excluded portion.

### 3.2 Physicochemical Properties

Steaks 1 and 2 exhibited significantly lower pH values than steak 12 (*P* < 0.05), although all samples remained within the normal pH range (Table 2). In addition, a significant linear decrease in pH was observed along the *Biceps femoris* muscle (*P* < 0.001). According to Kuffi et al. (2018), the pH is not uniformly distributed within the muscle postmortem, varying according to tissue depth due to the combined effects of cooling and ongoing metabolic processes. And this heterogeneity directly influences key quality traits, including texture, color, water‐holding capacity, and tenderness (Silva et al. 1999). The progressive decline in pH observed from proximal to distal regions in the present study reinforces the existence of metabolic and structural gradients along the *Biceps femoris* muscle.

**TABLE 2:** Least squares means for pH, sarcomere length, cooking loss, and Warner‐Bratzler shear force (WBSF) of *Biceps femoris* muscle.

Steak ^A^	pH	Laser sarcomere (µm)	Microscopy sarcomere (µm)	Cooking loss (%)	WBSF (kg)
1	5.79^a^	1.77^ab^	1.82^abc^	21.4^a^	4.28^d^
2	5.79^a^	1.76^ab^	1.79^abc^	22.3^a^	4.40^d^
3	5.78^ab^	1.83^ab^	1.87^ab^	21.4^a^	4.31^d^
4	5.77^ab^	1.97^a^	1.97^a^	22.4^a^	4.22^d^
5	5.76^ab^	1.75^b^	1.78^abc^	21.6^a^	4.30^d^
6	5.73^ab^	1.77^ab^	1.79^abc^	20.5^a^	4.26^d^
7	5.71^ab^	1.78^ab^	1.78^abc^	21.0^a^	4.69^cd^
8	5.75^ab^	1.83^ab^	1.84^abc^	21.6^a^	4.91^bcd^
9	5.70^ab^	1.69^b^	1.74^bc^	20.9^a^	5.67^abc^
10	5.73^ab^	1.62^b^	1.75^bc^	20.0^a^	5.89^ab^
11	5.71^ab^	1.70^b^	1.71^bc^	21.2^a^	6.07^a^
12	5.62^b^	1.64^b^	1.65^c^	21.8^a^	6.18^a^
SEM	0.033	0.041	0.045	0.697	0.315
*P*‐value	< 0.05	< 0.001	< 0.01	0.504	< 0.001
Pr > F linear	< 0.001	< 0.001	< 0.001	0.509	< 0.001
Pr > F quadratic	0.404	< 0.05	< 0.05	0.413	< 0.01

*Note*: The same letter in the same column are not significantly different (*P* < 0.05); ^A^Starting from the proximal end (steak 1) toward the distal end (steak 12).

Abbreviation: SEM, standard error of the mean.

**FIGURE 2:**
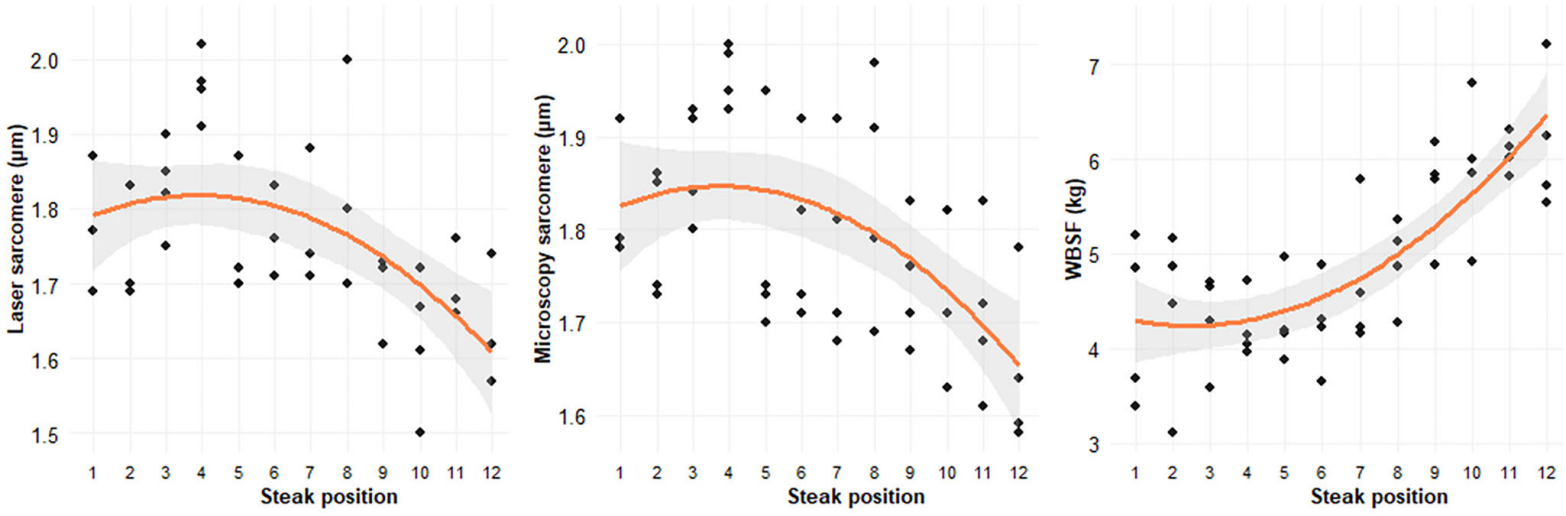
Significant quadratic models for different analyses conducted on steaks of the *Biceps femoris* muscle. Note: Steak position: starting from the proximal end (steak 1) toward the distal end (steak 12).

For sarcomere length determined by laser diffraction, steak 4 exhibited the greatest sarcomere length (1.97 µm), differing significantly from steak 5 (1.75 µm) and from the distal steaks of the *Biceps femoris* (steaks 9–12; 1.63–1.70 µm), whereas no significant differences were observed among the remaining steaks. A similar trend was observed using phase‐contrast microscopy, with steak 4 presenting the longest sarcomere length (1.97 µm) and steak 12 the shortest value (1.65 µm) (Table 2). Both techniques revealed significant linear and quadratic effects (*P* < 0.01). Sarcomere length remained relatively stable between steaks 1 and 3, increased to a peak at steak 4, and subsequently decreased from steaks 5 to 7. Although these intermediate values were numerically similar, a slight increase was observed at steak 8, followed by a consistent reduction toward the distal region (steaks 9–12) (Figure 2). Longer sarcomeres are generally associated with increased tenderness due to reduced actomyosin overlap and lower myofibrillar resistance (Battaglia et al. 2019; Ertbjerg and Puolanne 2017). These results therefore suggest that steaks from the proximal region, corresponding to the Picanha portion, are structurally predisposed to greater tenderness compared to steaks from the distal region (Coxão Duro).

No effect of steak position on cooking loss was observed (*P* > 0.05), with values ranging from 20.0% to 22.4% (Table 2). These results indicate that, despite the structural and compositional differences observed along the *Biceps femoris* muscle, the water‐holding capacity during thermal processing remained relatively constant across the evaluated anatomical regions. Cooking loss is influenced by several factors, including muscle pH, protein denaturation, intramuscular fat content, connective tissue characteristics, and the structural integrity of the muscle fibers (Bejerholm et al. 2014). The absence of significant differences suggests that the combined influence of these factors resulted in similar moisture losses during cooking, despite the progressive changes observed in collagen content, sarcomere length, and fat deposition along the muscle.

Cuts designated as “tender” must exhibit Warner–Bratzler shear force (WBSF) values ≤4.4 kg (ASTM 2011). In the present study, steaks 1 through 6 met the criteria for the “tender” classification, whereas steaks 7 through 12 fell outside this threshold and were therefore categorized as non‐tender. Steaks 11 and 12 exhibited significantly higher shear force values compared with steaks 1 to 8 (*P* < 0.05; Table 2). This pattern was further supported by significant linear and quadratic trends (*P* < 0.001 and *P* < 0.01, respectively). Shear force values remained relatively similar from steaks 1 to 6, followed by a slight increase at steak 7 and a progressive rise toward the distal region, reaching maximum values at steaks 11 and 12 (Figure 2). The quadratic response highlights that tenderness decline is not linear but accelerates in the distal portion of the muscle. This comportment is consistent with structural changes along the *Biceps femoris*, suggesting that proximal steaks possess more favorable sensory attributes compared to distal cuts. The reduction in tenderness in distal regions can be attributed to intrinsic structural and biochemical characteristics of the muscle. Starkey et al. (2016) demonstrated that the *Biceps femoris* exhibits higher collagen content in distal regions, which increases shear resistance and negatively impacts tenderness perception.

Total moisture content was lower in steak 2 compared with steak 10 (*P* < 0.05). An inverse pattern was observed for total fat content, with steak 2 showing higher total fat than steak 10 (*P* < 0.05). However, steak 5 did not differ from the other positions for either attribute (*P* > 0.05; Table 3). The inverse relationship between total moisture and total fat in meat is well established, reflecting the replacement of water by lipid within the muscle structure. Cuts with higher total fat concentrations are often associated with improved tenderness and greater sensory acceptability (Dunshea et al. 2021). This effect is primarily attributed to the role of fat in enhancing lubrication during chew, reducing perceived toughness, and contributing to a more favorable mouthfeel (Martinez et al. 2023). Therefore, the greater total fat concentration observed in the proximal portion of the *Biceps femoris* compared with the distal region represents a positive quality attribute, particularly in the context of premium cuts such as Picanha, where sensory appeal and gastronomic value are closely linked to fat content and eating experience.

**TABLE 3:** Least squares means for total moisture and fat contents of *Biceps femoris* muscle.

Steak ^A^	Total moisture (g.100g^−1^)	Total fat (g.100g^−1^)
2	73.7^b^	3.12^a^
5	74.3^ab^	2.56^ab^
10	74.5^a^	1.97^b^
SEM	0.209	0.156
P‐value	<0.05	<0.01

*Note*: The same letter in the same column are not significantly different (*P* < 0.05); ^A^Starting from the proximal end (steak 1) toward the distal end (steak 12).

Abbreviation: SEM, standard error of the mean.

The results for the different collagen fractions are presented in Table 4. Total collagen and insoluble collagen contents were lower in steaks 2 and 5 compared with steak 10 (*P* < 0.05), whereas steak 7 did not differ significantly from the other positions. Regarding soluble collagen, steaks 2, 5, and 7 showed the lowest values (0.28–0.37 mg.g^−1^), in contrast to steak 10, which presented the highest concentration (1.56 mg.g^−1^) (*P* < 0.05). These findings indicate a progressive gradient in collagen distribution along the *Biceps femoris* muscle, particularly noticeable around steak 7, suggesting a transitional zone between the proximal and distal regions. Rhee et al. (2004) quantified total collagen across different regions of the *Biceps femoris* muscle and reported higher values in the distal portion, consistent with those observed in the present study. When evaluating 11 beef cuts collectively, Rhee et al. (2004) reported significant correlations between collagen content and sensory attributes such as overall tenderness and perceived amount of connective tissue, as well as instrumental texture measurements. A similar pattern was observed when the *Biceps femoris* was evaluated independently, highlighting the substantial influence of collagen on final meat quality.

**TABLE 4:** Least squares means for total, soluble and insoluble collagen of *Biceps femoris* muscle.

Steak ^A^	Total collagen (mg.g^−1^)	Soluble collagen (mg.g^−1^)	Insoluble collagen (mg.g^−1^)
2	1.94^b^	0.33^b^	1.61^b^
5	2.23^b^	0.37^b^	1.86^b^
7	3.42^ab^	0.28^b^	3.14^ab^
10	6.77^a^	1.56^a^	5.21^a^
SEM	0.663	0.153	0.527
P‐value	< 0.001	< 0.001	< 0.01

*Note*: The same letter in the same column are not significantly different (*P* < 0.05); ^A^Starting from the proximal end (steak 1) toward the distal end (steak 12).

Abbreviation: SEM, standard error of the mean.

### 3.3 Histological Analysis

**FIGURE 3:**
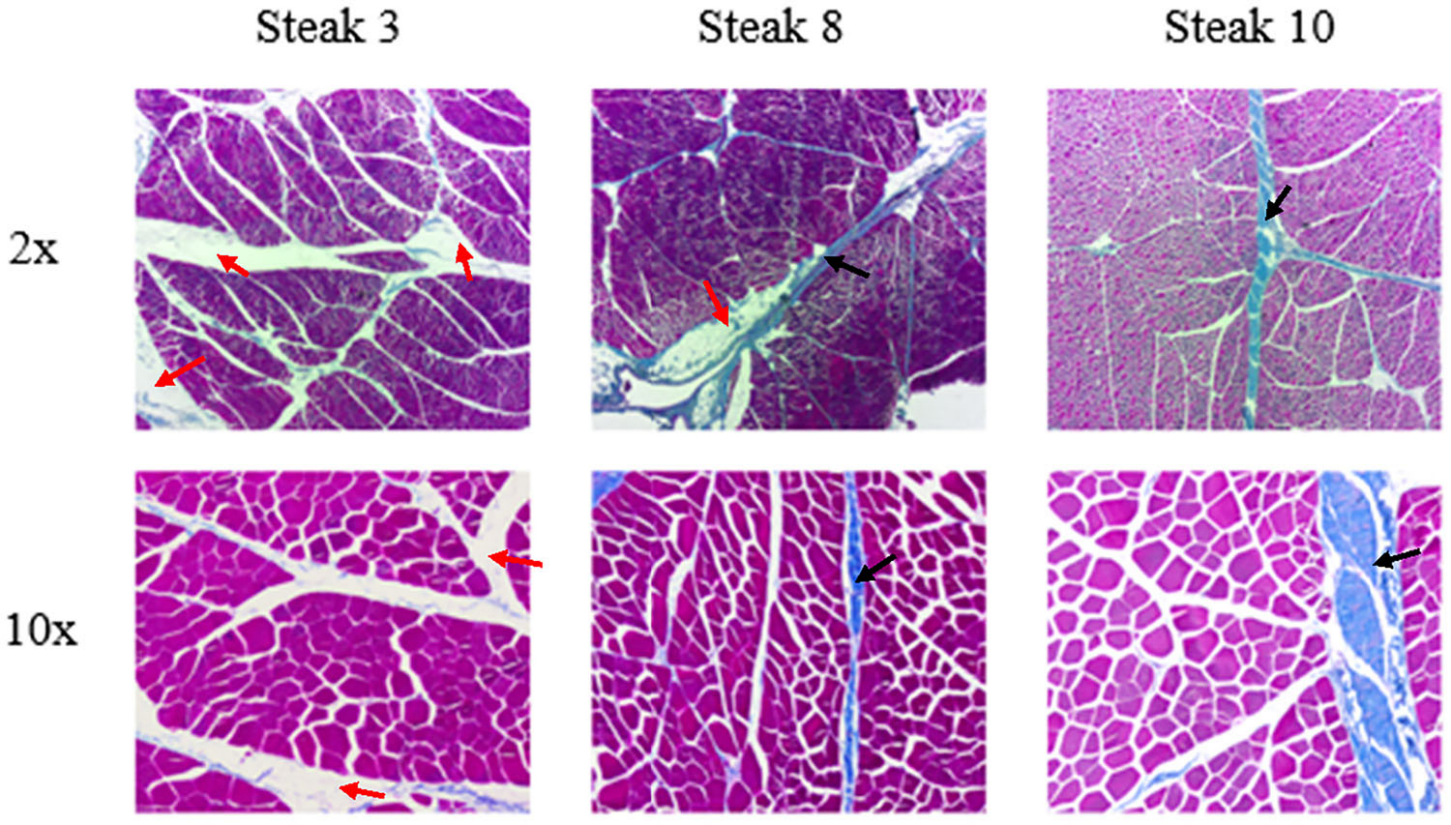
Histological micrographs of steaks 3, 8, and 10 at 2 × and 10 × magnifications of bovine *Biceps femoris* muscle. *Note*: Red arrows indicate fat cells, and black arrows indicate connective tissue structures. Steaks—starting from the proximal end toward the distal end.

Histological analysis was performed on samples 3, 8, and 10 with micrographs captured at 2× and 10× magnifications. The fields presented in Figure 3 were selected based on the morphological representativeness of the features that contribute to distinguishing between cuts, focusing on the relationship between muscle structures.

At 2× magnification, steak 3 showed the presence of fusiform and elongated bundles, with the cells within these bundles closely packed, making it difficult to separate them for counting cells per bundle. The bundles appeared loosely arranged in the field, with little or no connective tissue surrounding them. Thin structures (red arrow), rounded in shape and stained blue, resembling fat cells (adipocytes), were observed (Bancroft and Layton 2019). In the slides of steak 8, the bundles also appeared fusiform and elongated, with closely grouped cells, again hindering cell counting per bundle. The bundles appeared more compact compared to steak 3, and were surrounded by blue‐stained structures resembling connective tissue (black arrow). Between the bundles, structures resembling adipocytes (red arrow) were present, more concentrated in certain areas, rather than interspersed within the bundles, as observed in steak 3.

In contrast to steaks 3 and 8, the slides of steak 10 showed bundles with a geometric shape and well‐defined edges. Cells were grouped within the bundles, but spacing between them created an organized appearance between cells and bundles, facilitating counting and assessment of cell density per bundle. The bundles appeared firmer compared to samples 3 and 8 and were uniformly distributed in the field. Between the bundles, thick blue‐stained structures resembling connective tissue (black arrow) were observed.

At 10× magnification, steak 3 showed closely packed cells within the bundles and spaces filled with blue‐stained cells resembling adipocytes (red arrow) interspersed between bundles, giving the appearance of total fat. In steak 8, cell morphology showed changes compared to steak 3, with some regions similar to steak 3 and others displaying more geometric‐shaped cells that were less clustered, allowing better visualization and increased apparent cell density per field. The muscle structure in steak 10 displayed cells with an even more defined geometric shape and a more uniform distribution across the field. The space between cells allowed clearer visualization, unlike what was observed in the previous steaks.

### 3.4 Pearson's Correlation

Correlation analysis was performed on steaks 2, 5, and 10, in four *Biceps femoris* muscle, using the results obtained from pH, laser and phase contrast microscopy sarcomere length, WBSF, cooking loss, total moisture and fat content, and total, soluble, and insoluble collagen content (Table 5). Insoluble collagen showed a positive correlation with soluble collagen (*r* = 0.961; P ≤ 0.001) and with total collagen (*r* = 0.998; P ≤ 0.001). In the context of tenderness, all collagen fractions were positively and significantly correlated with shear force (WBSF). These results are supported by Nishimura (2010) and Purslow (2018), indicating that higher collagen concentrations, particularly in the insoluble form, increase the mechanical resistance of muscle fibers, resulting in tougher meat.

**TABLE 5:** Pearson correlation coefficients among physical and chemical analyses from steaks 2, 5 and 10 of *Biceps femoris* muscle.

	SC	TC	Total fat	Total moisture	Microscopy sarcomere	Laser sarcomere	WBSF	Cooking loss	pH
**IC**	0.961^***^	0.998^***^	−0.653^*^	0.320	−0.305	−0.435	0.815^**^	−0.549	−0.386
**SC**		0.978^***^	−0.606^*^	0.340	−0.324	−0.548	0.729^**^	−0.610^*^	−0.479
**TC**			−0.646^*^	0.327	−0.312	−0.466	0.800^**^	−0.569	−0.412
**Total fat**				−0.583^*^	0.404	0.591^*^	−0.766^**^	0.711^**^	0.549
**Total moisture**					0.031	−0.331	0.367	−0.305	−0.597^*^
**Microscopy sarcomere**						0.784^*^	−0.436	−0.004	0.219
**Laser sarcomere**							−0.543^*^	0.357	0.499
**WBSF**								−0.391	−0.298
**Cooking loss**									0.577^*^

*Note*: P‐value: *P < 0.05, **P < 0.01, ***P < 0.001.

Abbreviations: IC, Insoluble collagen; SC, Soluble collagen; TC, Total collagen; WBSF, Warner‐Brattzler Shear Force.

Sarcomere length measured by microscopy showed a significant positive correlation with laser‐measured sarcomere length (*r* = 0.784; P ≤ 0.05). A similar result was reported by Battaglia et al. (2019), who identified a correlation between methods for assessing tenderness and measuring sarcomere length using laser diffraction and phase‐contrast microscopy, validating both procedures. Laser‐measured sarcomere length was significantly negatively correlated with WBSF (*r* = –0.543; P ≤ 0.05), indicating that longer sarcomeres favor the production of more tender meat (Lepetit 2007).

A negative correlation was observed between total fat and collagen content, with significant negative correlations for insoluble collagen (*r* = –0.653; P ≤ 0.05), soluble collagen (*r* = –0.606; P ≤ 0.05), and total collagen (*r* = –0.646; P ≤ 0.05). This pattern suggests that cuts with higher fat content tend to have a lower proportion of connective tissue, contributing to greater tenderness, as fat acts as a separating element between muscle bundles and enhances positive sensory perception (Gardner et al. 2020). Total fat also showed a significant negative correlation with total moisture (*r* = –0.583; P ≤ 0.05), a relationship likely explained by volumetric replacement of water by fat in the muscle matrix. Moreover, total fat was negatively correlated with cooking losses (*r* = –0.766; P ≤ 0.01), indicating that higher lipid content contributes to reducing exudation during cooking, possibly due to a physical barrier to fluid loss and changes in thermal dynamics during cooking. The pH showed a significant negative correlation with total moisture (*r* = –0.597; *P* < 0.05). This inverse relationship between pH and total moisture may be associated with differences in water‐holding capacity, as meat with higher pH tends to retain more water bound to myofibrillar proteins but may have a lower percentage of free water, resulting in a reduced overall moisture content.

The data demonstrate that connective tissue structure, proximate composition, sarcomere length, and physicochemical properties of meat interact in a complex manner, primarily influencing tenderness. The association between collagen and toughness confirms the structural role of connective tissue in shear resistance, while the presence of fat contributes to tenderness and reduces cooking losses. Correlations between laser and microscopy‐measured sarcomere lengths, as well as between laser‐measured sarcomere length and tenderness, indicate that longer sarcomeres may contribute to tenderness, particularly when associated with lower collagen content. Overall, the observed trends reinforce that the presence of longer sarcomeres can be an influential factor in sensory quality, especially when combined with low collagen levels and higher fat content.

## 4 Conclusion

This present study demonstrates that the segmentation of the *Biceps femoris* into Picanha and Coxão Duro is supported by measurable compositional, mechanical, and structural differences along the muscle. Steaks located before to the “third vein” exhibited higher total fat, lower total and insoluble collagen, longer sarcomeres, and reduced shear force values, all attributes associated with greater instrumental tenderness and consequently improved sensory perception under dry‐heat cooking methods such as grilling on barbecue, for example. In contrast, distal steaks displayed progressively higher collagen concentrations and greater mechanical resistance, characteristics more compatible with alternative culinary applications requiring moist heat or extended cooking times. These findings suggest that the traditional industrial practice of delimiting Picanha is not only cultural or commercial but also reflects intrinsic anatomical and structural transitions within the muscle. By integrating muscle biology with culinary application, this study reinforces the importance of scientifically grounded cut standardization to ensure consistency in eating quality, preserve gastronomic identity, and support international commercialization of culturally beef cuts. However, future studies including different breeds, carcass weights, production systems, and slaughter ages are necessary to further validate the applicability of the proposed anatomical criterion.

## Author Contributions

**Isabela Benfica de Barros**: data curation, formal analysis, investigation, methodology, project administration, Writing – original draft, Writing – review and editing. **Jonatã Henrique Rezende‐de‐Souza**: formal analysis, investigation, visualization, writing – review and editing, writing – original draft, data curation, project administration. **Dyana Carla Lima Hargreaves Noguera**: investigation, writing – review and editing. **Gabriel Barão Arias Blanco**: data curation, formal analysis, investigation, writing – review and editing. **Cíntia Rizoli**: investigation, methodology. **Sílvio Roberto Consonni**: resources, supervision, investigation, visualization, methodology. **Sérgio Bertelli Pflanzer**: conceptualization, resources, supervision, investigation, visualization, methodology, project administration, writing – review and editing, writing – original draft.

## Conflicts of Interest

The authors declare no conflicts of interest

## Supporting information




**Supplementary Table S1**: jfds71424‐sup‐0001‐TableS1.docx
