# Supplementary material for: Technical Criteria for Delimiting the Picanha Beef Cut: A Preliminary Study
Source: J Food Sci. 2026 Sep 4;91(9):e71424. doi: 10.1111/1750-3841.71424 (PMC13544472; doi:10.1111/1750-3841.71424)
Supplement: Supplementary file 1 [file JFDS-91-0-s001.docx]

**Supplementary Table 1.** Marking (X) the first, second and third veins on cuts of beef from the B*iceps femoris* muscle.

| **Steak ^A^** | ***First vein*** | | | |  | ***Second vein*** | | | |  | ***Third vein*** | | | |
| --- | --- | --- | --- | --- | --- | --- | --- | --- | --- | --- | --- | --- | --- | --- |
|  | **Carcass ^B^** | | | |  | **Carcass ^B^** | | | |  | **Carcass ^B^** | | | |
|  | **1** | **2** | **3** | **4** |  | **1** | **2** | **3** | **4** |  | **1** | **2** | **3** | **4** |
| 1 | - | - | - | - |  | - | - | - | - |  | - | - | - | - |
| 2 | X | X | X | X |  | - | - | - | - |  | - | - | - | - |
| 3 | - | - | - | - |  | - | - | - | - |  | - | - | - | - |
| 4 | - | - | - | - |  | X | X | X | X |  | - | - | - | - |
| 5 | - | - | - | - |  | - | - | - | - |  | - | - | - | - |
| 6 | - | - | - | - |  | - | - | - | - |  | X | X | X | X |
| 7 | - | - | - | - |  | - | - | - | - |  | - | - | - | - |
| 8 | - | - | - | - |  | - | - | - | - |  | - | - | - | - |
| 9 | - | - | - | - |  | - | - | - | - |  | - | - | - | - |
| 10 | - | - | - | - |  | - | - | - | - |  | - | - | - | - |
| 11 | - | - | - | - |  | - | - | - | - |  | - | - | - | - |
| EP1 | - | - | - | - |  | - | - | - | - |  | - | - | - | - |
| 12 | - | - | - | - |  | - | - | - | - |  | - | - | - | - |
| EP2 | - | - | - | - |  | - | - | - | - |  | - | - | - | - |

Note: ^A^ Starting from the proximal end (steak 1) toward the distal end (steak 12). ^B^ The samples from the left and right sides of the same animal showed the same results. EP: Excluded portion (distal muscle portion excluded from the quality analyses because it belonged entirely to the outside round region and was not considered part of the anatomical transition between Picanha and Coxão Duro).
